# Diagnostic value of a CT-based radiomics nomogram for discrimination of benign and early stage malignant ovarian tumors

**DOI:** 10.1186/s40001-023-01561-1

**Published:** 2023-12-19

**Authors:** Jia Chen, Fei Yang, Chanzhen Liu, Xinwei Pan, Ziying He, Danhui Fu, Guanqiao Jin, Danke Su

**Affiliations:** 1 Department of Radiology, Guangxi Medical University Cancer Hospital, 71 Hedi Road, Nanning, Guangxi People’s Republic of China; 2 Department of Radiology, Guangxi Clinical Medical Research Center of Imaging Medicine, 71 Hedi Road, Nanning, Guangxi People’s Republic of China; 3 Department of Radiology, Guangxi Key Clinical Specialties, 71 Hedi Road, Nanning, Guangxi People’s Republic of China; 4 Department of Radiology, Guangxi Medical University Cancer Hospital Superiority Cultivation Discipline, 71 Hedi Road, Nanning, Guangxi People’s Republic of China; 5 Department of Clinical Medical, Guangxi Medical University, 22 Shuangyong Road, Nanning, Guangxi People’s Republic of China; 6 Department of Gynecologic Oncology, Guangxi Medical University Cancer Hospital, 71 Hedi Road, Nanning, Guangxi People’s Republic of China

**Keywords:** Radiomics nomogram, Malignant ovarian tumor, Computed tomography

## Abstract

**Background:**

This study aimed to identify the diagnostic value of models constructed using computed tomography-based radiomics features for discrimination of benign and early stage malignant ovarian tumors.

**Methods:**

The imaging and clinicopathological data of 197 cases of benign and early stage malignant ovarian tumors (FIGO stage I/II), were retrospectively analyzed. The patients were randomly assigned into training data set and validation data set. Radiomics features were extracted from images of plain computed tomography scan and contrast-enhanced computed tomography scan, were then screened in the training data set, and a radiomics model was constructed. Multivariate logistic regression analysis was used to construct a radiomic nomogram, containing the traditional diagnostic model and the radiomics model. Moreover, the decision curve analysis was used to assess the clinical application value of the radiomics nomogram.

**Results:**

Six textural features with the greatest diagnostic efficiency were finally screened. The value of the area under the receiver operating characteristic curve showed that the radiomics nomogram was superior to the traditional diagnostic model and the radiomics model (*P* < 0.05) in the training data set. In the validation data set, the radiomics nomogram was superior to the traditional diagnostic model (*P* < 0.05), but there was no statistically significant difference compared to the radiomics model (*P* > 0.05). The calibration curve and the Hosmer–Lemeshow test revealed that the three models all had a great degree of fit (All *P* > 0.05). The results of decision curve analysis indicated that utilization of the radiomics nomogram to distinguish benign and early stage malignant ovarian tumors had a greater clinical application value when the risk threshold was 0.4–1.0.

**Conclusions:**

The computed tomography-based radiomics nomogram could be a non-invasive and reliable imaging method to discriminate benign and early stage malignant ovarian tumors.

**Supplementary Information:**

The online version contains supplementary material available at 10.1186/s40001-023-01561-1.

## Background

Ovarian cancer is one of the three common malignant tumors of the female reproductive system. In 2018, there were about 295,414 new cases and 184,799 ovarian cancer-related deaths worldwide, while only about 25% of ovarian cancer patients were diagnosed at early stage [1]. Imaging examination is vital for tumor detection, localization, benign and malignant determination, staging evaluation of malignant tumors, and prognosis of patients with ovarian cancer. However, early stage ovarian malignancies (FIGO stage I/II) are relatively insidious and has no obvious clinical symptoms. In clinical practice, the experience and professional level of imaging departments and clinicians noticeably influence the early diagnosis of ovarian cancer. In particular, the imaging features of benign and early stage malignant ovarian tumors often overlap. To identify only basing on the clinical manifestations, tumor markers, and the traditional computed tomography (CT) manifestations recognized by the naked eye, is lacking repeatability and objectivity, and often fails to accurately identify the nature of the lesion.

Although pathological diagnosis is the gold standard for distinguishing benign and malignant tumors, it belongs to an invasive examination. Sampling can only reflect the local condition rather than the entire tumor, and there are limitations in operation and the risk of tumor dissemination. In China, as a routine diagnostic method for ovarian cancer, CT possesses the advantages of being non-invasive, fast, and better presentation of morphological characteristics of ovarian lesions. It has markedly contributed to the diagnosis, as well as determination of the treatment protocols and evaluation of therapeutic effects [2]. Radiomics has noticeably attracted radiologists’ attention in recent years. The tumor heterogeneity is assessed objectively and quantitatively by radiomics through extracting the developable high-dimensional imaging features (e.g., intensity, geometry, texture, etc.) from medical images, and then, using a series of statistical tools and algorithms to quantitatively analyze the extracted features [3, 4]. To date, radiomics has been widely used in the study of diverse types of cancer. Wei et al. [5] constructed a new radiomics model using imaging phenotype and clinical variables to predict the overall survival time (OS) of hepatocellular carcinoma (HCC) patients receiving stereotactic body radiation therapy (SBRT). Huang et al. [6] developed an ultrasound-based radiomics model to distinguish between sclerosing adenopathy (SA) and invasive ductal carcinoma (IDC) to avoid misdiagnosis and unnecessary biopsy. Ramtohul et al. [7] evaluated whether the radiological characteristics based on multi parameter dynamic enhanced MRI could help to distinguish the expression of HER2 in breast cancer. The results showed that the radiological characteristics and tumor descriptors from multi parameter dynamic enhanced MRI could predict different HER2 expressions in breast cancer with therapeutic significance. Kang et al. [8] demonstrated that a radiomics nomogram model based on CT images can predict the recurrence and metastasis of clear cell renal cell carcinoma.

In recent years, many studies have shown that radiomics nomograms can provide valuable and reliable information for ovarian tumors. The radiomic nomogram was first used in a 2016 study published in the Journal of Clinical oncology. The study combined selected radiomic features with clinically relevant risk factors to construct nomograms. This prediction model can effectively conduct preoperative personalized prediction for patients with colorectal cancer lymph node metastasis and build a stable and feasible prediction model [9]. Radiomic nomogram analysis can be performed on medical images from different modes, such as CT, MRI, and ultrasound. Pan et al. [10] constructed a combined nomogram model based on radiological and semantic features for preoperative classification of serous and mucinous pathological types in ovarian cystadenoma patients. Hu et al. [11] investigated a nomogram based on arterial phase CT radiomics features and clinical features to help distinguish primary and secondary ovarian cancer. Li et al. [12] explored the difference in the application potential of two-dimensional (2D) and three-dimensional (3D) radiomics models based on non-contrast CT scans in differentiating benign and malignant ovarian tumors. The results showed that 2D and 3D radiomics nomogram models had comparable diagnostic efficacy in the differential diagnosis of benign and malignant ovarian tumors. Li et al. [13] combined radiomic characteristics and clinical factors to construct a radiomic nomogram based on venous phase CT, which has good clinical application value in the differential diagnosis of ovarian cystadenoma and endometriosis cyst. Zhang et al. [14] constructed a nomogram model combining radscore and clinical features, which can be used to detect synchronous ovarian metastasis in female gastric cancer patients. To differentiate benign, borderline, and malignant ovarian serous tumors, Qi et al. [15] integrated novel radiomics signatures from ultrasound and clinical factors to create a nomogram, thereby reducing or avoiding the risk of biopsy and surgery. Yao et al. [16] constructed a clinical–radiomics nomogram, which applied ultrasound radiomics features to distinguish the histopathological types of EOC for the first time, helping gynecologists to identify the types of EOC noninvasively before surgery. Xu et al. [17] used the image nomogram based on diffusion weighted imaging (DWI) and apparent diffusion coefficient (ADC) to classify epithelial ovarian tumors, which has significant clinical significance. CT is the most important imaging method for assessing the extent of ovarian tumors, facilitating preoperative tumor staging and rational surgical planning [18]. However, there are few studies on CT-based radiomics in early ovarian malignancies. Therefore, the present study aimed to explore the diagnostic value of a CT-based radiomics nomogram for discrimination of benign and early stage malignant ovarian tumors.

## Methods

### Patients’ general data

The clinical and imaging data of 197 ovarian tumor patients, including 98 cases of benign ovarian tumors and 99 cases of early stage malignant ovarian tumors, who were admitted to the Affiliated Tumor Hospital of Guangxi Medical University (Nanning, China) from January 2015 to November 2021 and met the eligibility criteria were retrospectively collected.

#### Inclusion criteria

1) Patients with benign and early stage malignant ovarian tumors who were confirmed by surgery or pathological biopsy; 2) patients who had received plain CT (P-CT) and contrast-enhanced CT (CE-CT) scan before surgery or puncture. 3) Patients with no history of relevant treatments (e.g., adjuvant radiotherapy or chemotherapy) before CT examination; 4) image quality meets diagnostic requirements.

#### Exclusion criteria

1) Patients with incomplete CT images or with poor quality images due to severe artifacts; 2) patients with a history of other malignant tumors, in addition to ovarian cancer; 3) extensive cystic degeneration, hemorrhage or calcification (make up 50% or more of lesions). The included cases were randomly assigned into training data set (*n* = 137) and validation data set (*n* = 60) according to the ratio of 7:3.

### CT image acquisition

Siemens Sensation 64 or GE Discovery 750HD scanners were used to perform the CT scan. The CT scanning parameters were as follows: tube voltage of 120 kV, tube current of 210 mA, layer thickness and layer spacing of equally 5 mm, and matrix size of 512 × 512. Fasting was essential 8–10 h before the examination and drinking of moderate amounts of water before the scan was permitted to keep the patient's bladder to be properly filled. During the examination, the patient was placed in a supine position, and the scan range was from the iliac crest to the lower edge of the pubic symphysis. If a lesion was not included, the scan range could be expanded. Perform conventional P-CT scan first, and then, inject the contrast agent, non-ionic contrast material iohexol (320 mg I/mL; Yangtze River Pharmaceutical) 90–100 mL, through the antecubital vein at a rate of 3–3.5 mL/s for the CE-CT scan. The scanning phase included P-CT scan, venous phase (with a delay of 70 s) CE-CT, and three-dimensional reconstruction of the venous phase was performed.

### Analysis of imaging features and radiomics analysis

#### Analysis of imaging features

Two board-certified radiologists with experience of more than 5 years of experience in gynecolgical tumor evaluated the P-CT and CE-CT images of each patient, including the size, location, shape, edge, degree of enhancement (10 HU (mild enhancement) < the difference in CT value of the lesion before and after enhancement ≤ 20 HU; 20 HU (moderate enhancement) < the difference in CT value of the lesion before and after enhancement ≤ 30 HU; and the severe enhancement was defined as the difference in CT value of the lesion before and after enhancement > 30HU), the existence of calcification, gas, necrosis, metastasis, invasion of neighboring organs, etc. Any discrepancy was resolved through a consensus-based discussion between two radiologists and a senior radiology chief physician (with 18 years of gynaecologic CT experience) made the final judgment.

#### Image segmentation

**Fig. 1:**
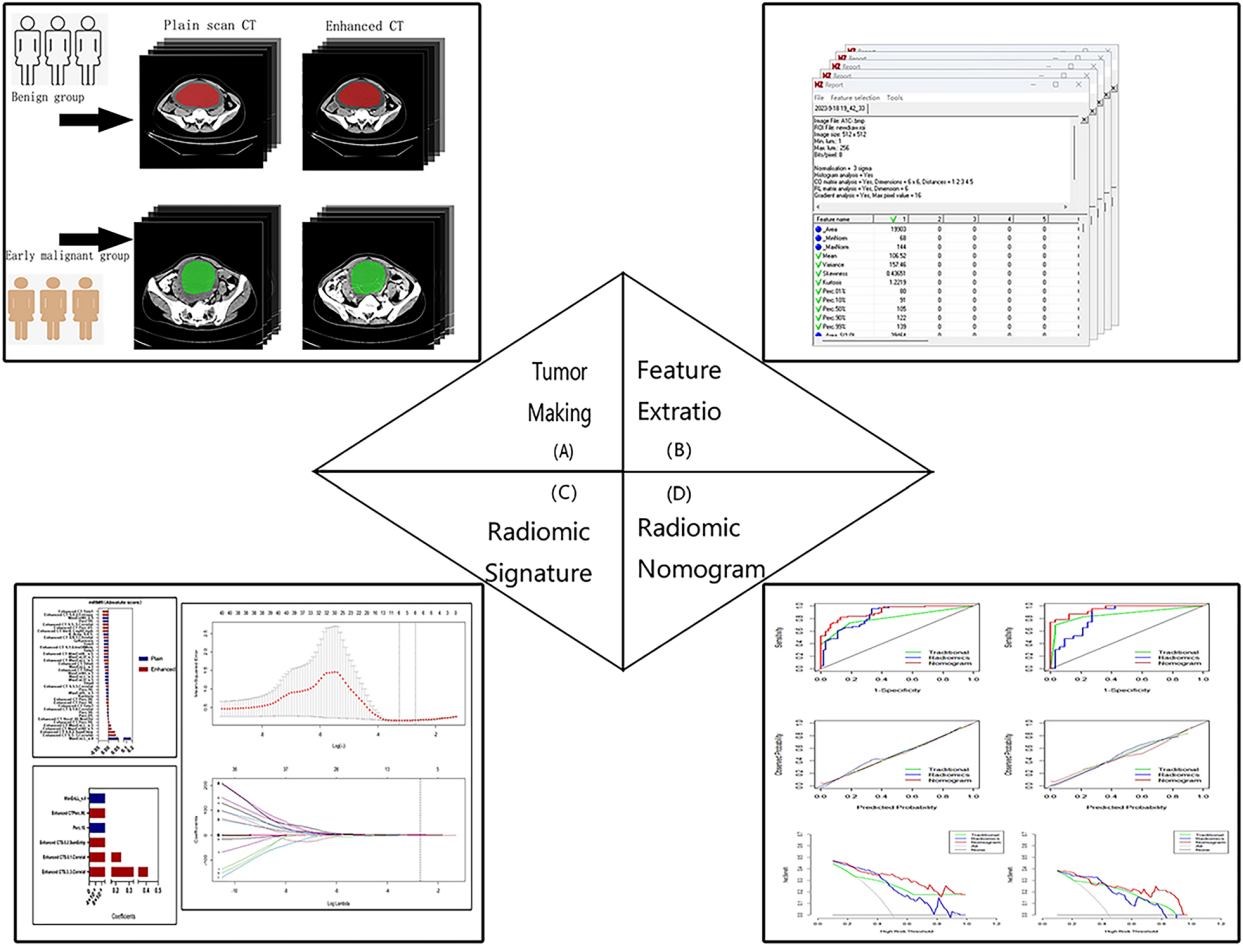
Study flowchart. The study flowchart includes 4 main steps: the first step was the acquisition of CT images and the drawing of the ROI of the primary lesion. The second step was the feature extraction from images of plain CT scan and contrast-enhanced CT scan. The third step was data analysis, including multivariate logistic regression analysis of traditional diagnostic factors and dimensionality reduction analysis of radiomics features; the fourth step was model construction and analysis of diagnostic efficacy

Transfer the largest layer of the lesion in BMP format from the PACS system to the Mazda post-processing software (Version 4.6, The Technical University of Lodz, Institute of Electronics, http://www.eletel.p.lodz.pl/mazda/). The images were randomly assigned to the two radiologists mentioned above, who independently segmented the ROI without knowing the patient's pathological results. All ROIs were reviewed by the senior radiology chief physician mentioned above. Regions of interest (ROIs) was drawn around tumor lesions on the software, and avoided delineation of organs, such as blood vessel and intestines as much as possible. The outer edge of the ROI was about 2 mm within the edge of the tumor to avoid partial volumetric effects. Physicians drew ROIs on each patient’s P-CT image and on the largest lesion in CE-CT image. As CE-CT image was clearer, first, the ROI on the CE-CT image was drawn. The ROIs should include the entire lesion as much as possible, and the selected ROIs should be saved. The P-CT imports the ROI to keep consistency with the ROI of CE-CT. The study flowchart is shown in Fig. 1.

#### Feature extraction

To minimize the influence of brightness and contrast variation in each ROI, a method in MaZda that normalizes image intensities in the range μ ± 3SD (μ, gray-level mean; SD, standard deviation) was used for gray-level normalization. A total of 302 textural features of all the ROIs could be calculated from the gray-level histogram, gray-level co-occurrence matrix (GLCM), the gray-level run-length matrix (GLRLM), the absolute gradient (GRA), the autoregressive model (ARM), and the wavelet transform (WAV) method.

#### Feature selection

Two feature selection methods (max-relevance and min-redundancy (mRMR) and the least absolute shrinkage and selection operator (LASSO)) were used to select the extracted radiomics features. First, the mRMR method was used to eliminate redundant and irrelevant features. Subsequently, the LASSO regression analysis was employed, and the LASSO regression model was selected through tenfold cross-validation to obtain the optimal hyperparameter λ value. At the optimal λ value, features with non-zero coefficients were used to calculate the radiomics score (Radscore). The Radscore of each patient was calculated based on images of P-CT and CE-CT. The Wilcoxon test was used to compare differences in the Radscore between benign and early stage malignant ovarian tumors in the training and the validation data sets. The predictive performance of the model could be evaluated using the receiver operating characteristic (ROC) curve analysis.

#### Construction of the radiomics model

In the present study, the radiomics model was established based on features extracted from images of P-CT and CE-CT, and the ROC curve analysis was employed to assess the predictive performance of these two models for discriminating benign and malignant ovarian tumors. The DeLong test was used to compare differences between the ROC curves plotted based on images of P-CT and CE-CT. Accordingly, the best radiomics model was selected to construct a radiomics nomogram.

#### Construction of the radiomics nomogram

The univariate analysis was carried out on CA125, HE4, tumor size ≥ 7 cm, ascites, and Radscroe of each patient, and independent predictors were selected for differentiating benign and malignant ovarian tumors; then, the radiomics nomogram was constructed through multivariate logistic regression analysis via combining radiomics features and independent clinical risk factors, while the traditional diagnostic model was established based on the independent clinical risk factors; these two models all were verified in the validation data set; the calibration curve was used to evaluate the predictive performance of the radiomics nomogram, and the Hosmer–Lemeshow test was used to analyze the fit to evaluate the calibration ability of the radiomics nomogram. The ROC curve analysis was utilized to evaluate the diagnostic efficacy of the radiomics nomogram; the decision curve analysis (DCA) was used to predict the clinical application value of the model.

### Data processing and statistical analysis

Statistical analysis was performed using the R 3.5.1 programming language (www.R-project.org) and the SPSS 23.0 software (IBM, Armonk, NY, USA). The participants were randomly assigned into training cohort (TC, *n* = 137) and validation cohort (VC, *n* = 60) at a ratio of 7:3. The obtained ROC curve and area under the curve (AUC) were used to evaluate the diagnostic efficacy of the model. The DeLong test was used to compare the diagnostic efficacy between models. The DCA was employed to evaluate the clinical application value of the combined predictive model. Categorical variables were compared by the Chi-squared (χ2) test, and continuous variables were compared by the independent-sample *t* test and the Wilcoxon test.

The mRMR method was used to remove redundant and irrelevant textural features in the TC group. The “glmnet” package utilized the LASSO algorithm on the features with the largest predictive performance to further select the optimal subset, and to construct the radiomics model. The Radscore of each patient was calculated using the linear combination of textural features selected by the LASSO and the weighting coefficient corresponding to each feature. The “pROC” package was used to draw ROC curves to evaluate the predictive performance of the model. The “ModelGood” package was employed to calibrate the model. A predictive model based on traditional diagnostic factors and Radscore was constructed through univariate and multivariate logistic regression analyses. The “rms” package was utilized to construct a nomogram. The “rmda” package was used to perform the DCA to evaluate the clinical applicability of the model. The stability and practicability of the corresponding model in the VC group was verified. *P* < 0.05 was considered statistically significant.

## Results

### Patients’ general data

Pathological results showed that among 197 patients with ovarian cancer, there were 98 cases of benign ovarian tumors and 99 cases of early stage malignant ovarian tumors, with an average age of 45.10 ± 15.02 years. The main clinical data and the results of CT texture analysis are shown in Table 1. The four indicators of CA125, HE4, maximum diameter of tumor, and ascites in the early stage malignant group were greater than those in the benign group, and the difference was statistically significant (*P* < 0.05); there were no statistically significant differences in age, menopausal status, CA199 level, regardless of consideration of a multinodular cyst, peritoneal thickening, a clear tumor boundary, or the degree of enhancement, between the two groups (*P* > 0.05). According to the ratio of 7:3, 137 and 60 cases were randomly assigned into TC and VC groups, respectively. There was no statistically significant difference in the mentioned four indicators between the training data set and the validation data set (*P* > 0.05).

**Table 1:** Comparison of clinical characteristics of benign and early malignant ovarian tumor training set and validation set

Indicator	Benign	Early malignant	*P* value	Training	Validation	*P* value
	(*n* = 98)	(*n* = 99)		(*n* = 137)	(*n* = 60)	
Age*	42.5 ± 14.5	47.6 ± 13.2	0.080	43.8 ± 14.6	47.8 ± 12.4	0.087
Menopause			0.050			0.061
No	67 (68.4)	51 (51.5)		88 (64.2)	30 (50.0)	
Yes	31 (31.6)	48 (48.5)		49 (35.8)	30 (50.0)	
Histology						0.766
Benign						
Serous	33 (33.7)			23 (16.8)	10 (16.7)	
Mucinous	21 (21.4)			15 (10.9)	6 (10.0)	
Endometrioid	7 (7.1)			5 (3.6)	2 (3.3)	
Teratoma	34 (34.7)			24 (17.5)	10 (16.7)	
Other	3 (3.1)			2 (1.5)	1 (1.7)	
Malignant						
Epithelial		62 (62.6)		43 (31.4)	19 (31.7)	
Sex cord stromal		22 (22.2)		15 (10.9)	7 (11.7)	
Embryonal		10 (10.1)		7 (5.1)	3 (5.0)	
Other		5 (5.1)		3 (2.3)	2 (3.2)	
CA125			0.000			0.409
≤ 35 µ/mL	78 (79.6)	26 (26.3)		75 (54.7)	29 (48.3)	
> 35 µ/mL	20 (20.4)	73 (73.7)		62 (45.3)	31 (51.7)	
CA199			0.312			0.528
≤ 37 µ/mL	77 (78.6)	77 (77.8)		101(73.7)	46 (76.7)	
> 37u/mL	19 (19.4)	22 (22.2)		36 (26.2)	14 (23.3)	
HE4			0.000			0.138
≤ 120 pmol/L	97 (99.0)	57 (57.7)		113 (82.5)	41 (68.3)	
> 120 pmol/L	1 (1.0)	42 (42.3)		24 (17.5.0)	19 (31.7)	
Maximum diameter			0.000			0.318
> 7 cm	45 (45.9)	14 (14.1)		44 (32.1)	15 (25.0)	
≤ 7 cm	53 (54.1)	85 (85.9)		93 (67.9)	45 (75.0)	
Ascites			0.000			0.551
Yes	28 (28.6)	67 (67.7)		68 (49.6)	27 (45.0)	
No	70 (71.4)	32 (32.3)		69 (50.4)	33 (55.0)	
Bilateral			0.055			0.251
Yes	46 (46.9)	60 (60.6)		70 (51.1)	36 (60.0)	
No	52 (53.1)	39 (39.4)		67 (48.9)	24 (40.0)	
Multilocular			0.075			0.186
Yes	91 (92.9)	84 (84.8)		119 (86.9)	56 (93.3)	
No	7 (7.1)	15 (15.2)		18 (13.1)	4 (6.7)	
Peritoneal thickening			0.063			0.683
Yes	16 (16.3)	27 (27.3)		31 (22.6)	12 (20.0)	
No	82 (83.7)	72 (72.7)		106 (77.4)	48 (80.0)	
Boundary			0.124			0.257
Clear	38 (38.8)	26 (26.3)		41 (29.9)	23 (38.3)	
Intervenient	32 (32.7)	40 (40.4)		53 (38.7)	19 (31.7)	
Obscure	28 (28.5)	33 (33.3)		43 (31.4)	18 (30.0)	
Enhancement			0.323			0.338
Mild	26 (26.5)	34 (34.3)		38 (27.7)	22 (36.7)	
Moderate	39 (39.8)	35 (35.4)		54 (39.4)	20 (33.3)	
Severe	33 (33.7)	30 (30.3)		45 (32.8)	18 (30)	

Data are numbers of lesions, with percentages in parentheses. Categorical variables were compared using the χ2 test or Fisher exact test. *Data are Means ± standard deviations and compared using the two-sample *t* test

### Feature selection

**Fig. 2:**
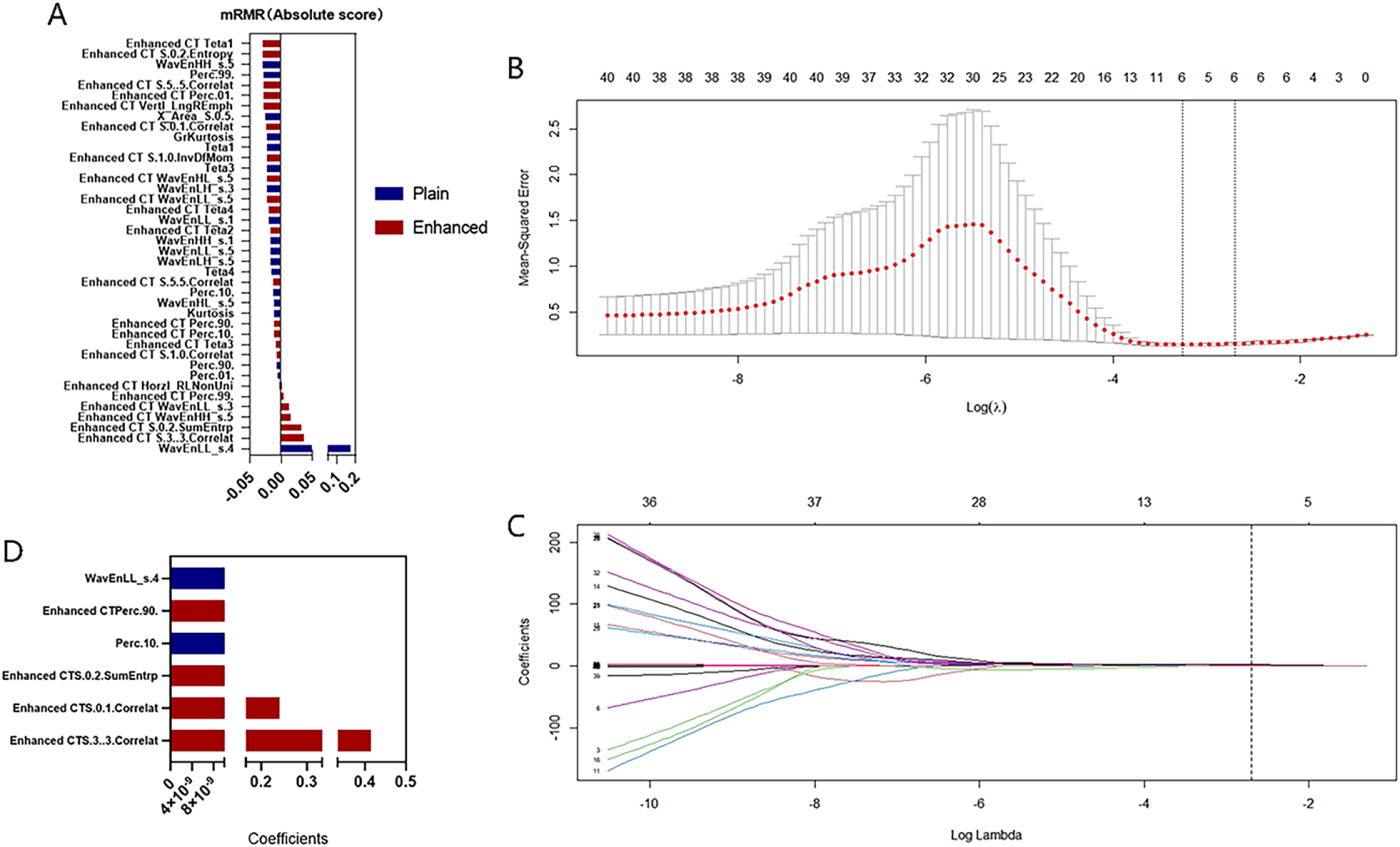
Feature dimensionality reduction analysis. **A** Features were ranked according to their mRMR (maximum correlation and minimum redundancy) scores. The top 20 features were selected using the mRMR algorithm. **B** Selection of the tuning parameter (Lambda) in the LASSO model using tenfold cross-validation. Binomial deviances from the LASSO regression cross-validation model were plotted as a function of log (Lambda). The dotted vertical line at the right was drawn at the optimal value based on the minimum criteria and the 1-standard error rule (the 1-SE criteria). An optimal Lambda value of 0.067 with log (Lambda) = − 1.174 and 6 non-zero coefficients were selected. **C** Profiles of the LASSO coefficients for the 6 texture features. The vertical line was drawn at a value selected from the log (λ) sequence using tenfold cross-validation. Six features of non-zero coefficients are shown. **D** Selected radiomic features and corresponding coefficients

In the present study, 302, 302, and 604 radiomics features were selected from P-CT scan, CE-CT scan, and combined scan (P-CT and CE-CT) images for each ROI, respectively (Additional file 1: Table S1). First, the mRMR method was used to select 20, 20, and 40 features (Fig. 2A), further optimized via the LASSO algorithm, and 2, 5, and 6 features were finally selected (Fig. 2B, and C). The ROC curve analysis was used to evaluate the predictive performance of the diagnostic model based on the P-CT, CE-CT, and combined scan features in the training data set and validation data set for predicting benign and malignant ovarian tumors, respectively. The results are presented in Table 2. Delong test showed that the difference in AUC value between the P-CT and CE-CT was not statistically significant (*P* = 0.123). The diagnostic efficiency of the combined scanning was higher than the other two methods, and the difference was statistically significant (*P* = 0.011). Therefore, features from the combined scanning were selected to construct a radiomics model (Fig. 2D).

**Table 2:** Evaluation results of image omics model in training set and validation set

Model	Group	Sensitivity (%)	Specificity (%)	Threshold	AUC (95% CI)
Plain	Training	0.953	0.682	− 0.529	0.861 (0.797–0.924)
	Validation	0.815	0.700	0.061	0.833 (0.731–0.935)
Enhanced	Training	0.938	0.864	− 0.595	0.954 (0.918–0.990)
	Validation	0.885	0.800	− 0.291	0.901 (0.823–0.980)
Both	Training	0.989	0.848	− 1.364	0.974(0.948–1.000)
	Validation	0.923	0.867	− 0.304	0.921(0.850–0.991)

AUC: Area under the curve

### Predictive performance of the radiomics model

The radiomics score (Radscore) of each patient was calculated as follows: Radscore = (0.4152847) × F1 + (0.2397519) × F2 + (0.060411) × F2 + (0.08415373) × F3 + (0.00604111) × F4 + (0.000097118440) × F5 + (0.0000001028209) × F6.

**Fig. 3:**
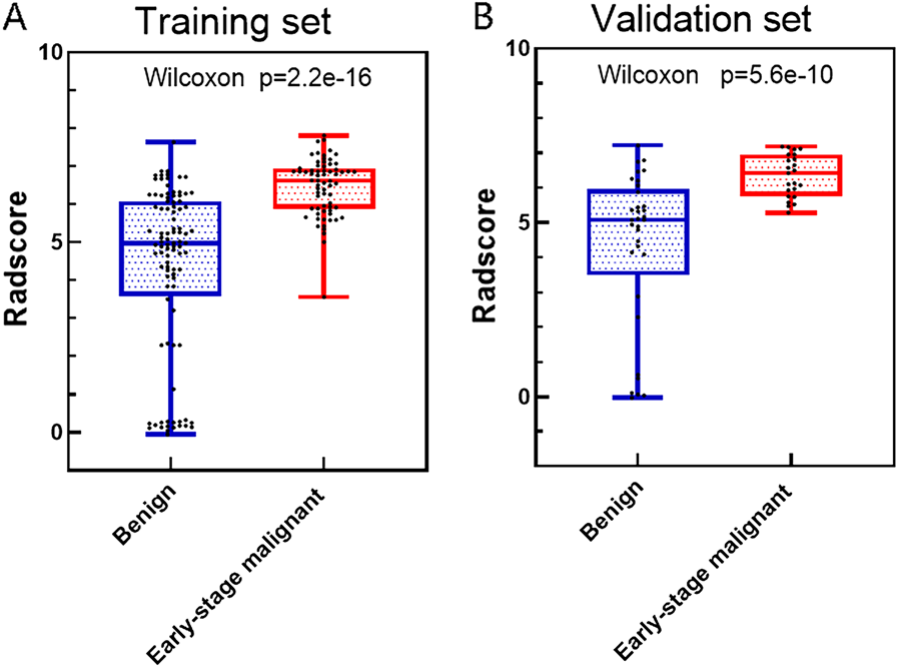
Comparison of the Radscore for benign and early stage malignant ovarian tumors on the training and validation sets

The results of the Wilcoxon test showed that the Radscore in the early stage ovarian malignant tumor group was higher than that in the benign group (Fig. 3), and the difference was statistically significant (*P* < 0.05), indicating that the Radscore was correlated with benign and early malignant ovarian cancer. In the TC group, the AUC value was 0.856, and the sensitivity and specificity were 0.955 and 0.667, respectively; in the VC group, the AUC value was 0.843, and the sensitivity and specificity were 0.957 and 0.727, respectively (Table 3).

**Table 3:** Comparison of radiomics models in training set and validation set

Group	Sensitivity	Specificity	Threshold	AUC (95% CI)
Training	0.955	0.667	0.383	0.856 (0.792–0.920)
Validation	0.957	0.727	0.317	0.843 (0.741–0.946)

AUC: Area under the curve

### Screening of predictive risk factors for benign and malignant ovarian tumors

**Fig. 4:**
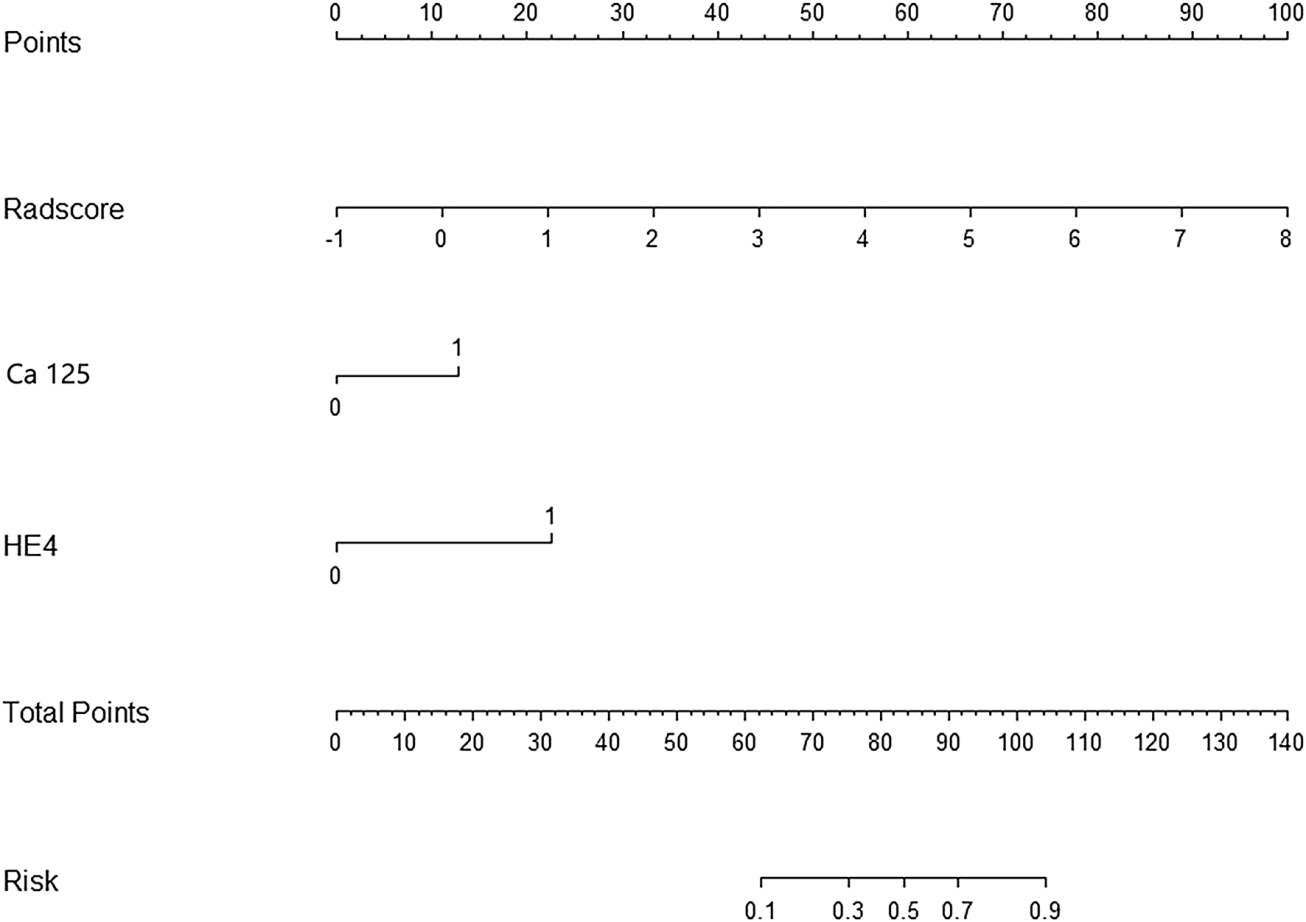
Radiomics nomogram for predicting benign and early stage malignant ovarian tumors

The results of univariate regression analysis showed that CA125, HE4, and maximum tumor diameter could be potential risk factors for predicting early stage malignant ovarian tumors (*P* < 0.10). The multivariate logistic regression and stepwise regression were then performed to obtain 2 predictive factors (CA125, HE4) related to the identification of benign and early stage malignant ovarian tumors, and the corresponding traditional diagnostic model was established. The results of multivariate logistic regression analysis showed that CA125, HE4, and Radscore were independent risk factors (*P* < 0.05), and they were employed to construct a radiomics nomogram (Fig. 4) to predict the nature of ovarian tumors (Table 4).

**Table 4:** Logistic regression analysis of clinical factors predicting benign and early stage malignant ovarian tumors

Clinical factors	Univariate logistic regression analysis		Multivariate logistic regression analysis	
	OR (95% CI)	*P* value	OR (95% CI)	*P* value
CA125	2.636 (0.996–6.977)	0.051*	0.261 (0.114–0.597)	0.001**
HE4	10.246 (1.136–92.395)	0.038*	0.069 (0.008–0.568)	0.013**
Maximum diameter	2.422 (0.903–6.500)	0.079*	0.427 (0.176–1.038)	0.060
Ascites	2.049 (0.816–5.146)	0.127		
Radscord			3.042 (1.771–5.228)	0.000**

*P < 0.10, **P < 0.05: included in the radiomics nomogram

### Predictive performance of the radiomics nomogram

**Fig. 5:**
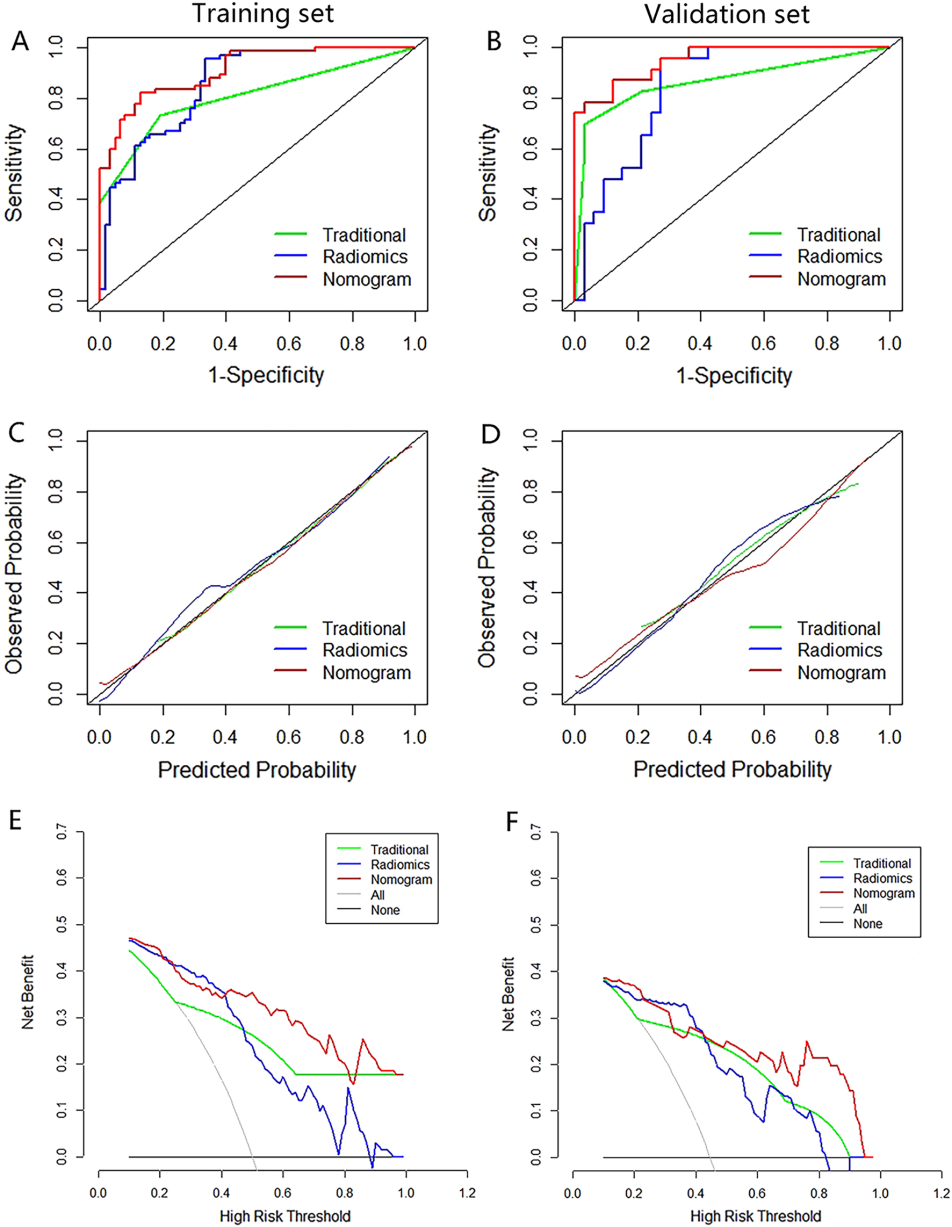
**A**, **B** ROC analysis of the traditional, radiomics algorithm and radiomic nomogram in predicting benign and early stage malignant ovarian tumors. **C**, **D** Calibration curve of the radiomics nomogram. **E**, **F** Decision curve of the radiomics nomogram

In the present study, the diagnostic performance of the radiomics model, the traditional model, and the nomogram model in predicting benign and early stage malignant ovarian tumors was compared (Fig. 5A, B and Table 5). The DeLong test showed that the difference in AUC value between the constructed nomogram model and the radiomics model, between the constructed nomogram model and the traditional diagnostic model, and between the radiomics model and the constructed nomogram model was statistically significant (*P* < 0.05).

**Table 5:** Diagnostic efficacy of the three groups of models in the training set and validation set

Model	Group	Sensitivity	Specificity	Threshold	AUC (95% CI)
Traditional	Training	0.731	0.810	0.459	0.807 (0.732–0.883)
	Validation	0.739	0.788	0.417	0.780 (0.651–0.909)
Radiomics	Training	0.955	0.667	0.383	0.856 (0.792–0.920)
	Validation	0.957	0.727	0.317	0.843 (0.741–0.946)
Nomogram	Training	0.821	0.873	0.566	0.910 (0.862–0.957)
	Validation	0.870	0.818	0.420	0.913 (0.841–0.985)

AUC: Area under the curve

The calibration curve and the Hosmer–Lemeshow test revealed that the difference between the predicted and the actual observed values of the three models in the training and validation data sets was not statistically significant (*P* > 0.05), indicating that there was no deviation fitting (Fig. 5C, D). The DCA curve showed that when the high-risk threshold was greater than 0.4, the net return rate of the nomogram model in the TC and VC group were higher than that in the radiomics model and the traditional diagnostic model (Fig. 5E, F).

## Discussion

The prognosis of ovarian malignant tumors depends on early stage diagnosis, surgical treatment, and postoperative systemic treatment. Therefore, the early and accurate identification of benign, malignant, and aggressive lesions is vital for the selection of an appropriate treatment option and prediction of prognosis of patients with ovarian cancer. However, the current routine gynecological examinations, traditional imaging features, and tumor markers all have certain difficulties in the qualitative diagnosis of early stage ovarian tumors. Therefore, how to use non-invasive methods to improve the diagnostic accuracy of early stage ovarian cancer has always been a hot topic for gynecologists. In this study, we provided three diagnostic models: a radiomics model constructed with optimal features, a traditional model that combines clinical manifestations, tumor markers, and traditional CT manifestations recognized by the naked eye, and a nomogram model that combines important traditional factors and radiomics features.

Radiomics extracts deep information that cannot be recognized by the human eye through in-depth exploration of high-dimensional features of CT images, and then, quantitatively analyzes the tumor heterogeneity, which can reflect tumor information more objectively and comprehensively [19]. In recent years, radiomics has been used to non-invasively identify benign and malignant ovarian tumors [20, 21], as well as for the purposes of histological grading [22], evaluation of molecular typing [23, 24], assessment of efficacy [25], and prediction of metastasis [26] and prognosis [27, 28]. At present, there is no relevant research on the use of CT imaging to distinguish benign and early stage malignant ovarian tumors, and research have mainly concentrated on the contrast-enhanced CT or MRI.

P-CT refers to scanning without intravenous injection of iodine containing contrast agent, while CE-CT refers to scanning under intravenous injection of iodine containing contrast agent. P-CT can provide basic anatomical structure information of the examination area, but usually cannot distinguish between benign and malignant lesions. CE-CT can clearly display the location, shape, range, internal components, blood supply, and presence or absence of metastasis of ovarian tumors. It is the preferred imaging examination method for preoperative FIGO staging and treatment planning of ovarian tumors. P-CT and CE-CT can provide different information, but both are indispensable components of ovarian tumors CT examination. Before performing enhanced scanning, P-CT scan is necessary. The differentiation of ovarian tumor tissues is associated with the characteristics of gray value. The radiomics of the P-CT scan has a potential value in the classification of ovarian tumor tissues. Therefore, utilization of the P-CT scan for tumor identification is logical in theory, and its working principle is easier than the CE-CT [22]. Previous studies [22, 26] have also shown that the radiomics model based on the P-CT images has a high diagnostic efficiency, which could be significant for the identification and prediction of ovarian benign and malignant tumors. Compared with P-CT scans, CE-CT scans can provide more valuable data and more comprehensively reflect the heterogeneity of tumors; nevertheless, the CE-CT is susceptible to the subjective influences of the contrast agent itself and an operator’s experience, and the results may be biased [29]. A previous research [26] showed that a nomogram based on venous CT radiomic has a promising efficacy in predicting lymph node metastasis in high-grade serous carcinoma. Hence, in the present study, we used the P-CT and CE-CT to identify further accurately benign and early stage malignant ovarian tumors, so that patients can receive earlier and more personalized treatment options without increasing the economic burden. In this study, a total of 604 quantitative texture features were extracted from P-CT and CE-CT images. Table 2 provides the diagnostic performance of the model constructed based on these radiomics features. The results showed that the AUC values of 1.00–0.90 for the combined diagnosis were evaluated as excellent [30], but the features were too redundant. Therefore, this article reduces the dimensionality of the combined features and ultimately obtains 6 optimal features, including 2 features from P-CT and 4 features from CE-CT, for Radscore calculation to identify benign and early malignant ovarian tumors.

In our study, a total of 6 radiomics features were obtained using the combined scanning, including S (0, 1) Correlat, S (3, 3) Correlat, Perc. 10%, Perc. 90%, S (0, 2) SumEntrp, and WavEnLL_s-4. S (0,1) Correlat and S (3,3) Correlat were correlated together, belonging to the characteristics of the GLCM. The correlation describes the degree of similarity of the GLCM in the row and column directions. When the correlation value is relatively uniform, the degree of similarity in the rows and columns is large [31]. S (0,2) SumEntrp indicates the sum of entropy, which is also a feature of the GLCM, and it could be used to describe the degree of textural complexity. The larger the entropy value, the more uneven the texture of the studied image [32]. Perc.10% and Perc.90% belong to the histogram feature parameters, and the histogram parameters are based on the distribution of the voxel intensity of the pixel distribution in the CT image to reflect the tissue heterogeneity. The larger the value, the more obvious the tumor heterogeneity [33]. WavEnLL_s-4 is the low-frequency wavelet coefficient, which is the low-frequency component of the wavelet transformation model, highlighting the low-spatial frequency component, and it is a high-order feature. Wavelet transform is an image processing technique using a combination of high-frequency and low-frequency bandpass filters to decompose an image to obtain important information that may be hidden from the image [34]. At present, high-order statistics are rarely used in radiomics, and the clinical significance of the parameters still needs to be further explored. The histogram of the Radscore values of the 6 optimal radiomics features shows that the Radscore values of the benign group are lower than those of the early malignant group, indicating that the tumor heterogeneity is more significant in the early malignant group. Table 3 shows that the radiomics model constructed using the 6 optimal radiomics features has high AUC values, sensitivity, specificity, and accuracy, indicating its very good diagnostic performance (AUC0.90–0.80) [30]; the AUC value in the training set (0.856) is slightly higher than the value in the validation data set (0.843), indicating that the radiomics model has strong generalization ability.

Univariate analysis showed that among the traditional diagnostic factors, Ca125, HE4, and the largest tumor diameter were correlated with benign and early malignant ovarian tumors (*P* < 0.1). Multivariate logistic regression analysis revealed that imaging features have a certain diagnostic value for benign and early stage malignant ovarian tumors, while they cannot be independent predictors. It was speculated that CT imaging features overlap between benign and early stage malignant ovarian tumors. The independent predictors of early stage malignant tumors were Ca125 and HE4 (*P* < 0.05), which was consistent with the results of higher preoperative levels of Ca125 and HE4 in patients with ovarian malignant tumors in a previous study [35]. Therefore, based on Ca125 and HE4, the present study constructed a traditional diagnostic model for ovarian benign and early stage malignant tumors. Compared with traditional diagnostic models, the radiomics model in the validation set increased AUC by 6.3% (AUC, 0.843 vs. 0.780, *P* = 0.435), but the difference was not significant. Compared with traditional models, the radiomics model has higher sensitivity (0.955 vs. 0.731) and lower specificity (0.667 vs. 0.810), indicating fewer missed diagnoses and ensuring diagnostic quality. However, the degree of intention was relatively low, and it was easy to be misdiagnosed; if these two methods are combined, they can compensate for their respective shortcomings and make the diagnosis to be more accurate.

Furthermore, we combined the traditional diagnostic model (Ca125 and HE4) and the radiomics model to construct a nomogram, which easy-to-use and showed an excellent predictive performance in both the training and the validation data sets. In the validation data set, using the traditional diagnostic model, the radiomics model, and the nomogram, the AUC values for predicting benign and early stage malignant ovarian tumors were 0.780, 0.843, and 0.913, respectively, which were all greater than 0.7, demonstrating that the models have a good diagnostic value [30], and the nomogram has the highest predictive capability. Compared with traditional diagnostic models and radiomics models, the nomogram showed an increase in AUC of 10.3% (0.910 vs. 0.807, *P* = 0.000) and 5.4% (0.910 vs. 0.856, *P* = 0.012) in the training data set, respectively; in the validation data set, AUC increased by 13.3% (0.913 vs. 0.780, *P* = 0.003) and 7.0% (0.843 vs. 0.913, *P* = 0.108), respectively. This indicates that compared to radiomics models, the nomogram has higher performance in the training data set, but weaker generalization ability, and there is no statistically significant difference in the validation data set; compared with traditional diagnostic models, nomograms exhibit higher performance in both training and validation data sets, and the differences are statistically significant. The robustness is verified through validation data sets. To evaluate the clinical usefulness of the radiomics nomogram, decision curve analysis (DCA) was applied in this study, which is a novel method to calculate the net benefit at various threshold probabilities to insight clinical consequences. DCA revealed that the radiomics nomogram has a greater clinical value in the discrimination of benign and early stage malignant ovarian tumors within the threshold range of 0.4–1.0, confirming a promising clinical utility. Our study found that the radiomics nomogram model outperformed in diagnostic accuracy, which consistent with a recently published study that arterial phase CT imaging feature and clinical feature to distinguish primary and secondary ovarian cancer. The results of that research showed that the combination of clinical factors and arterial phase CT radiomics features was more efficient than using them alone [11]. Another study used radiomics to identify benign and malignant bone tumors, which showed that the radiomics nomogram model included clinical and radiomics features performed well in both training and validation data sets. The AUC, DCA, and net reclassification improvement (NRI) revealed that compared with the clinical model, the radiomics nomogram model exhibited a better diagnostic performance, and it has a greater clinical net benefit than the pure clinical and radiomics model [36].

The limitations of this study should be pointed out. First, the sample size was small, the pathological type of distribution was unbalanced, and the results need to be independently verified by a larger sample size and involvement of multiple centers. Second, the thickness of the scan was 5 mm, which was theoretically correct for the second order and high level of the extracted lesions. The value of first-order image features might have an impact. Finally, this was a retrospective study, and there was an inevitable selection bias.

## Conclusions

In summary, the radiomics model could discriminate benign and early stage malignant ovarian tumors, and the radiomics nomogram model that combines traditional and radiomics models shows the best diagnostic performance, which is valuable for developing personalized treatment plans.

### Supplementary Information

**Additional file 1**.  Radiomics features extracted from ROI

## Data Availability

The data sets used and/or analysed during the current study are available from the corresponding author on reasonable request.

## Abbreviations

CT: Computed tomography
P-CT: Plain computed tomography
CE-CT: Contrast-enhanced computed tomography
ROIs: Regions of interest
FIGO: Federation international of gynecology and obstetrics
GLCM: Gray-level co-occurrence matrix
GLRLM: The gray-level run-length matrix
GRA: Absolute gradient
ARM: Autoregressive model
WAV: Wavelet transform
mRMR: Max-relevance and min-redundancy
LASSO: Least absolute shrinkage and selection operator
ROC: Receiver operating characteristic
DCA: Decision curve analysis
TC: Training cohort
VC: Validation cohort
AUC: Area under the curve
NRI: Net reclassification improvement
